# Characteristics of participants in the national research mentoring network studies

**DOI:** 10.1017/cts.2025.10133

**Published:** 2025-08-26

**Authors:** So Hee Hyun, Emma Dums, Fátima Sancheznieto, Kimberly Spencer, Julie M. Hau, Jenna Griebel Rogers, Christine Pfund

**Affiliations:** Institute for Clinical and Translational Research (ICTR), University of Wisconsin–Madison, Madison, WI, USA

**Keywords:** Mentorship, national research Mentoring Network (NRMN), biomedical workforce, demographics, mentor training

## Abstract

**Introduction:**

This paper presents a descriptive analysis of common data collected across 11 independent studies in the National Research Mentoring Network (NRMN) from 2019 to 2024, focusing on participant demographics and participation in training programs prior to NRMN.

**Methods:**

Analyses focused on data from 6,197 survey responses collected primarily at baseline. Descriptive analyses examined participants’ demographic characteristics (gender, combined race/ethnicity, disability, parent/guardian education, and career stage) and participation in training programs prior to NRMN.

**Results:**

The majority of respondents were female (70%). Most respondents identified as White (46%), Black (23%), Asian (18%), and Hispanic (17%). Most respondents (91%) did not report a disability. In terms of career stage, 55% were undergraduates. Sixty-three percent reported that their parent or guardian had completed a bachelor’s degree. Regarding participation in training programs prior to NRMN, 60% had participated in mentor training, and 62% reported involvement in research training activities such as workshops, field experiences, and conferences. Patterns of participation in prior mentorship or research program varied across demographic characteristics.

**Conclusions:**

The NRMN common data reveal the backgrounds of over 6,000 participants engaged in mentorship intervention studies across the biomedical workforce. The dataset includes participants from diverse demographics and career stages with varying levels of participation in prior mentor training and exposure to research training programs. This extensive dataset provides a valuable opportunity to explore the long-term impact of mentorship on the biomedical workforce in future research.

## Introduction

The advancement of the biomedical research workforce depends on the inclusion of individuals from diverse backgrounds [1–7]. Understanding the factors that contribute to the experiences, success, and persistence of individuals from these groups is essential for fostering a more equitable and innovative scientific community. The National Research Mentoring Network (NRMN), a key initiative within the National Institutes of Health’s Diversity Program Consortium (DPC), was established to support the career development of individuals from diverse backgrounds in biomedical research, with a focus on mentorship and professional development [8].

In its first phase (2014–2019), the NRMN demonstrated significant success in building a network of mentors and mentees from a variety of disciplines, impacting over 10,000 individuals (www.nrmnet.net) and over 7,000 mentors and mentees engaged in research mentorship training or intensive grantsmanship coaching [9–14]. Phase II (launched in 2019) built on this foundation by investigating longer-term outcomes and identifying effective mentorship strategies, particularly for diverse populations. This phase reimagined the NRMN as a consortium consisting of one coordination center (the NRMN CC), a resource center (the NRMN RC) [15] and 11 independent research studies that focused on mentorship, career development, and professional networking (Table 1). These studies shared a common goal of exploring how specific mentorship practices influence career outcomes for diverse groups in biomedical research.

**Table 1.** Overview of National Research Mentoring Network (NRMN) phase II studies: study title, principal investigator, and brief description

Study title	Principal Investigator name and institution	Study description
Boosting Mentor Effectiveness in Training of Research Scientists	Vineet Arora, University of Chicago	Used a randomized trial to test the effectiveness of virtual mentor training on women and minority medical student persistence in research careers during the required scholarly concentration programs at eight medical schools that participate in the Scholarly Concentrations Collaborative.
Impact of Culturally Aware Mentoring Interventions	Angela Byars-Winston, University of Wisconsin–Madison	Trained 600 faculty mentors in culturally aware mentoring practices to facilitate the academic success of doctoral students in biomedical disciplines and included research on outcomes for individual faculty mentors and factors for organizational change in departmental culture.
Building a Diverse Biomedical Workforce Through Communication Across Difference	Carrie Cameron, University of Texas MD Anderson Cancer Center	Tested the effects of an intervention on communicating across various dimensions of difference in dyads of summer undergraduate research programs (SURP) and their PhD or postdoctoral junior mentors (summer supervisors) by conducting novel communication workshops at 4 U54 cancer centers.
Studying Inclusive Mentor Networks to Diversify the Biomedical Workforce	Mica Estrada, University of California, San Francisco	A scalable social inclusion intervention study that involved hundreds of faculty and thousands of undergraduate students, as an add-on to the Tiny Earth program,[37] to inform the field of mentorship science and enhance future mentorship programs with the aim of broadening participation in the STEM fields.
Peer Group Mentoring for Racially Underrepresented Early Career Biomedical Researchers	Susan Girdler, University of North Carolina at Chapel Hill	Examined the benefits of adding psychosocial, peer group assisted mentoring on topics including microaggressions and imposter syndrome, to typical skills-based mentoring for underrepresented biomedical postdoctoral or junior faculty researchers.
Exploring the Intersection of Social Capital, Mentorship, and Networking	Manoj Mishra, Alabama State University	Implemented a mixed experimental design, with random selection and assignment of participants to Active Intervention (test) and Control Intervention (control) groups, focused on freshmen undergraduates at three historically black colleges and universities in the South, with the aim of increasing persistence, engagement and science identity of the test group resulting in the successful transition to the next career stage.
Testing Developmental Network Coaching for Diverse Early-Stage Investigators	Elizabeth Ofili, Morehouse School of Medicine	Randomized, controlled study that tested a structured grant writing plus developmental network coaching intervention study for early-stage investigators, compared to grant writing coaching alone.
Enhanced Grant Writing Coaching Intervention for a Diverse Biomedical Workforce	Kolawole Okuyemi, Indiana University	Built on the investigator’s previous NRMN-funded research,[24] provided grant writing skills training to underrepresented junior investigators to enhance their productivity and independence, looking at coaching dosage and engagement.
Career Advancement and Culture Change in Biomedical Research: Group Peer Mentoring	Linda Pololi, Brandeis University	Randomized controlled trial group of a peer mentoring program for mid-career physician-scientists and Ph.D. scientists engaged in biomedical research in U.S. medical schools and teaching hospitals.
Building Up a Diverse Workforce for Biomedical Research	Doris Rubio, University of Pittsburgh	Tested the effectiveness of an intervention, which includes coursework, networking, mentoring, and collaborative sessions for underrepresented minorities, in a cluster randomized controlled trial at 25 academic institutions.
Effectiveness of Innovative Research Mentor Interventions among Underrepresented Minority Faculty in the Southwest	Akshay Sood, University of New Mexico	Investigated mentoring training approaches and inter-institutional mentoring support networks.

Some titles listed in this table are adjusted. Source of information: National Research Mentoring Network. NRMN U01 Studies as a Part of NRMN Phase II. https://nrmnet.net/nrmn-u01-studies-as-a-part-of-nrmn-phase-ii/.

### NRMN independent research studies

Each of the 11 NRMN Phase II research studies consisted of a unique set of research aims, intervention models, recruitment strategies, and data collection designs. All studies led interventions for multiple groups of participants, with more than half of the studies leading interventions for ten or more separate cohorts. Most interventions were conducted virtually between October 2020 and June 2024. Recruitment strategies varied across studies, with some employing site-specific recruitment to target participants from particular institutions and others using national recruitment strategies to reach participants from a wide range of geographic regions and institutions. At least one study utilized both site-specific and national recruitment strategies. Study participants ranged from undergraduate students to faculty from hundreds of universities and institutions across all US regions, including public and private universities, minority-serving institutions, and research institutions. The data collection design for each study varied; however, all studies administered baseline surveys (pre-intervention) and post-surveys (post-intervention). The majority of studies also employed longitudinal study designs, with survey timepoints ranging from 3 months post- to 48 months post-intervention. Survey measures were determined by each study, although all studies agreed to use a set of community-agreed-upon common measures. More information on study intervention timelines and data collection design can be found on the NRMN Phase II Data Site (https://nrmndata.sites.wisc.edu/). A brief description of each study is also included in Table 1, and details on research outcomes can be found in emerging publications [16–25].

### NRMN common measures

Prioritizing the use of common metrics across studies, the NRMN Coordination Center (CC) worked closely with the 11 research teams to build a collection of common measures aligned with the DPC Hallmarks of Success [26]. These common measures included a set of required demographic, background, and career progression measures, along with additional measures, some of which were related to the other Hallmarks of Success. The establishment of these common metrics supported consistency across the studies, facilitating comparisons and comprehensive analyses of participant data. A list of the measures is available on the NRMN Phase II Data Site (https://nrmndata.sites.wisc.edu/).

With these common measures in place, the data collection process yielded a rich, diverse dataset that provides a unique opportunity to explore the factors influencing career development and mentorship effectiveness for diverse groups in biomedical research across participated in the 11 studies. As a result of the standardized approach to common measure data collection across these studies, the compiled common measures dataset has the capacity to deepen our understanding of how mentorship practices can influence the career progression of participants from diverse backgrounds and in varied contexts.

### Descriptive analysis of NRMN common data

This paper presents descriptive analyses of the common measures data collected from participants across the NRMN Phase II studies, with a particular focus on demographic characteristics and participation in training programs prior to NRMN. The dataset includes key demographic variables such as gender, combined race/ethnicity, disability, parent/guardian education, and career stage, as well as information on prior mentor training and participation in mentorship or research programs. The complete common measures dataset with survey data across all timepoints consists of 15,928 survey responses, including 6,360 baseline responses and 9,568 follow-up responses. The follow-up responses correspond to the same individuals who completed the baseline surveys. This paper primarily focuses on baseline data to provide a descriptive overview of NRMN Phase II participants and their prior participation in mentor training and other programs with a research training component. The NRMN multi-study common measures dataset represents one of the largest and most diverse training populations ever assembled across biomedical research, offering a unique opportunity to uncover patterns and generate insights that were previously difficult, if not impossible, to obtain. These insights are crucial for understanding the diverse needs of the biomedical research workforce and identifying factors that contribute to the persistence and success of all individuals. Ultimately, these data have the potential to shape future research and practices that foster a more inclusive and equitable biomedical research workforce.

## Method

### Data collection tracking

To support systematic and consistent tracking of research activities across the 11 NRMN studies, tracking spreadsheets were created for each study. These spreadsheets were used to collect detailed information on various aspects of the research, including project design, recruitment strategies, participant enrollment, intervention timelines, survey design, survey administration, and the usage of common measures. Both the NRMN CC and designated members from each study’s research team had access to the tracking spreadsheets, enabling collaboration, sharing of updates, and confirmation of the accuracy of the recorded information.

The NRMN CC managed an Institutional Review Board (IRB) protocol at the University of Wisconsin–Madison to securely transfer data from the participating research studies. Research teams were instructed to upload common measure survey data periodically throughout the grant into a secure folder in University of Wisconsin–Madison Box, a cloud storage platform. The first set of common measures data was received in March 2021, with final data requests sent to the research teams in April 2024 – two months before the official end of the 5-year grant period. Data that could not be transferred by the requested deadline from studies with no-cost extensions, which extended their interventions and data collection beyond the grant’s official end date, were excluded from the analyses described here.

Upon receipt, the data files were reviewed by NRMN CC researchers for consistency and quality. Any data that did not correspond to the agreed-upon common measures were excluded from the dataset to maintain focus and coherence. Once verified, the common measures data from all participating studies were compiled into a unified dataset, which serves as the foundation for the subsequent analyses. The dataset was structured to ensure compatibility across studies, facilitating meaningful comparisons and enabling a robust exploration of trends and patterns within the participant data.

### Data processing

**Figure 1.**
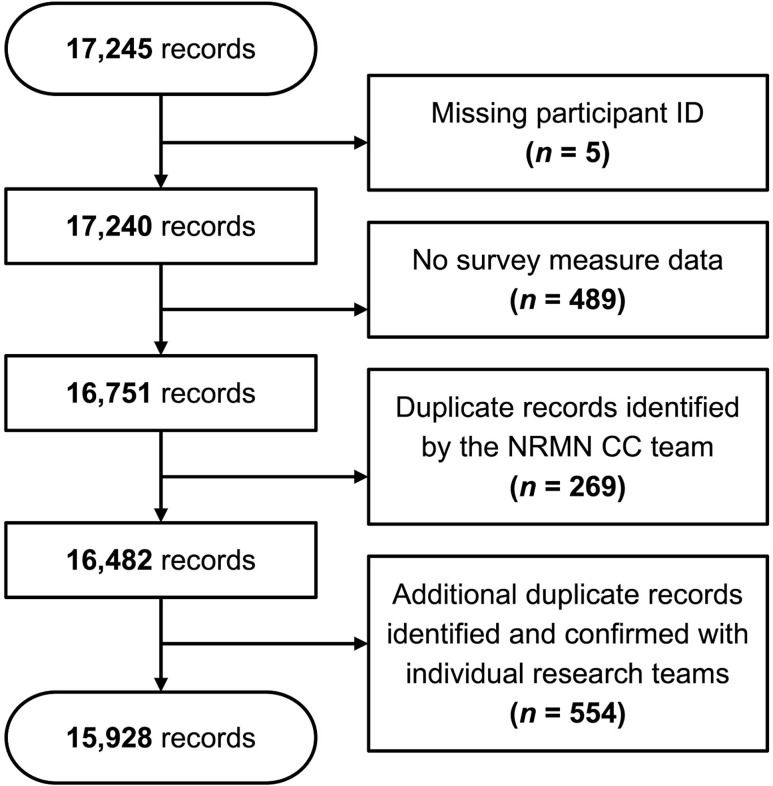
Initial data processing of the National Research Mentoring Network (NRMN) common data. Of 17,245 records, 494 did not have a participant ID or did not have any data across all survey measures, 269 were duplicates identified by our team, and 554 were duplicates identified by other NRMN research study teams. The resulting dataset contains 15,928 records, with 6,360 records from baseline surveys and 9,568 from follow-up surveys. While follow-up responses are shown here to represent the full scope of data collected, the analyses presented in this paper focus primarily on baseline data. In a few cases where demographic data were collected at follow-up, those responses were used to supplement baseline information. This figure reflects the dataset version compiled for the manuscript analyses; additional refinements will appear in future versions prepared for additional analyses and public data sharing.

The unified dataset initially contained 17,245 records from the 11 research teams. After applying exclusion criteria to remove duplicates and invalid cases (such as missing participant IDs, cases without common measure data, and unresolved duplicates), the subsequent dataset included 15,928 survey responses – 6,360 baseline responses and 9,568 follow-up responses (Figure 1). While the analyses presented here primarily focused on the baseline data, Figure 1 displays the total number of survey responses, including both baseline and follow-up responses, to reflect the full scope of data collected across the NRMN Phase II studies. Demographic variables were generally collected at baseline; however, for a limited number of teams that collected demographic information at follow-up, those responses were incorporated in the dataset to provide an in-depth overview of participants across all NRMN studies. This figure represents the dataset version compiled specifically for the analyses in this manuscript; further refinements will be incorporated in future versions prepared for additional analyses and public data sharing.

Duplication checks were conducted using exact matches of participant IDs and study-specific identifiers, allowing us, to the best of our ability, to identify and remove duplicates across cohorts within the same study. However, it was not feasible to detect all duplicate participation across all 11 NRMN studies, particularly if individuals moved between institutions or studies and were assigned different IDs. Because shared identifiers were not available across studies, some individuals may have contributed more than one record to the dataset. This is a known limitation of the dataset and highlights the importance of consistent identifiers in multi-study data collection efforts.

Additionally, not every case included data for all common measures. In some cases, the research teams employed slightly different sets of measures based on their specific study aims and designs, but also there were incomplete surveys, multiple versions of surveys, skipped questions, and variations in whether certain questions were required. In this paper, we primarily focused on analyzing cases from surveys including demographic information and participation in training programs prior to NRMN that were generally consistent with the common measures. We also included select data such as single-select demographic items that differed slightly from the common measures but were important to retain because they provided valuable demographic information about the participants in NRMN Phase II studies. We considered these data sufficiently comparable for the purpose of this paper while acknowledging their differences. The specific criteria for exclusion are detailed in the figure legend of Figure 1, which further explains the initial data cleaning and consolidation process.

### Descriptive analyses

In this paper, we describe the demographic characteristics of NRMN Phase II participants, assessing five key measures: gender, combined race/ethnicity, disability, parent/guardian education, and career stage. We also present participation in training programs prior to NRMN, specifically analyzing two key experience measures: prior mentor training and prior mentorship or research program engagement as examples of what type of exploratory analyses can be done with these types of data.

#### Demographic information

To provide an overview of NRMN Phase II participant characteristics, we selected five key demographic measures from the dataset based on the number of responses available, consistency in measure usage, and representation across studies. Participants with data for any of the five measures were included in the demographic analysis, for a total of 6,197 participants across all 11 studies. The NRMN CC developed a career stage variable based on information provided by the research teams. While most teams shared detailed career stage data for individual participants, some focused on specific target populations, such as general faculty or undergraduate students. The data collected from each team were integrated to categorize participants’ career stages. It is important to consider that not all of these individuals provided data for all five of the variables we have chosen to report and that the sample size varies by measure. Additionally, we identified differences in question wording, scales, and response formats. For example, at least one study collected data on combined race/ethnicity and gender in a single-select format as opposed to the multi-select format used in the original common measure. While we included these single-select responses for the purpose of this paper, further consideration may be needed for use of the data in other contexts.

#### Experience: prior mentor training

Baseline data on prior mentor training were collected from five research teams, with a total of 1,405 participants responding to a baseline survey that included the common measure on prior mentor training participation. Of these, 1,177 participants (approximately 83.8%) provided responses to the prior mentor training measure. The remaining 228 participants (about 16.2%) did not respond to the item and were excluded from the analysis.

#### Experience: prior mentorship or research program

Baseline data on the prior mentorship or research program engagement measure (“Have you ever been a part of any of the below programs focusing on research training? Select all that apply”) were available from eight research teams, with a total of 3,130 participants responding to a baseline survey that included this common measure. Of these, 2,204 participants (approximately 70.4%) reported participation in at least one program, while 926 participants (29.6%) did not report prior program participation and were excluded from the analysis. The final analysis included the 2,204 participants who reported prior involvement in at least one mentorship or research program. Subgroup analyses were also performed using available demographic data on gender, combined race/ethnicity, and career stage. Data on gender were available for 2,133 of the respondents, while combined race/ethnicity data were available for 2,140 participants. Career stage information was provided for 2,198 participants.

Descriptive statistics, including frequencies and measures of central tendency, were computed using IBM SPSS Statistics (Version 29) to summarize the demographic characteristics and prior training experience of the participants. Frequency analyses assessed the distribution of key demographic measures and prior training participation within the sample. Since the primary goal of this paper is to provide a descriptive profile of the participants based on the NRMN common data, we highlight general trends and patterns in the participants’ demographic characteristics and prior training participation using descriptive statistics rather than conducting formal statistical tests to answer specific research questions.

## Results

### Part 1. Demographics

Of the 5,791 respondents to the gender measure, a majority (70.4%) identified as female (Table 2). Of those who responded to the race/ethnicity question (*n* = 5,813), 560 participants (9.6%) reported more than one race/ethnicity. The most common racial/ethnic groups reported by participants were White (46.0%, *n* = 2,676), Black or African American (23.4%, *n* = 1,363), Asian (18.3%, *n* = 1,064), and Hispanic, Latinx, or Spanish origin (16.8%, *n* = 977). Most respondents (91.4%) did not report a disability. While 91.7% of respondents selected the “None” response option for the disability measure (as shown in Table 2), a small number of respondents who selected both “None” and a disability were included in the data. When asked about parent/guardian education, 63% of the respondents reported that one or more of the adults who raised them completed a bachelor’s degree.

**Table 2.** Respondent characteristics

Characteristic (*n* = respondents)	*n*	%
Gender* (*n* = 5,791)		
Female	4,079	70.4
Male	1,653	28.5
Nonbinary	56	1.0
Transgender	15	0.3
Other (please specify)	19	0.3
Combined race/ethnicity* (*n* = 5,813)		
White	2,676	46.0
Black or African American	1,363	23.4
Asian	1,064	18.3
Hispanic, Latinx, or Spanish origin	977	16.8
Middle Eastern or North African	148	2.5
American Indian or Alaska Native	83	1.4
Native Hawaiian or Other Pacific Islander	44	0.8
Some other race, ethnicity, or origin	105	1.8
Disability* (*n* = 5,142)		
None	4,714	91.7
Learning/cognitive disability	112	2.2
Blind/visually impaired	93	1.8
Vocal/speech disability	89	1.7
Physical/orthopedic disability	54	1.1
Deaf/hard of hearing	25	0.5
Other (please specify)	112	2.2
Parent/Guardian bachelor’s degree (*n* = 5,291)		
Yes	3,333	63.0
No	1,958	37.0
Career stage (*n* = 6,173)		
Undergraduate student	3,381	54.8
Faculty	1,932	31.3
Medical student	355	5.8
Postdoctoral fellow	346	5.6
Graduate student	80	1.3
Other	79	1.3

* Respondents were asked to select all categories that apply.

Participants represented a broad range of career stages, with the majority being undergraduate students (54.8%), followed by faculty (31.3%), medical students (5.8%), and postdoctoral fellows (5.6%). The faculty category included participants across all stages of their academic careers, from early-career faculty (e.g., assistant professors) to mid-career and senior faculty (including associate and full professors). A detailed breakdown of faculty stages was not provided for all participants and thus excluded.

### Part 2. Prior training experience

#### Experience: prior mentor training

The prior mentor training survey question asked, “Have you ever participated in any mentor training?” with yes/no response options. Of the 1,177 respondents, 60.4% (*n* = 711) reported having participated in some form of mentor training, while 39.6% (*n* = 466) indicated they had not.

#### Experience: prior participation in mentorship or research program

Of the 2,204 respondents who reported prior participation in at least one mentorship or research program, the most frequently reported programs were research training activities (e.g., workshops, field experiences, and conferences), with 61.5% (*n* = 1,355) of participants indicating involvement (Table 3). Other commonly reported program engagement included summer research programs (46.2%, *n* = 1,018), career advancement programs (39.1%, *n* = 861), and academic advising and support programs (35.3%, *n* = 778). Fewer respondents reported participation in postgraduate or graduate programs (26.2%, *n* = 578), undergraduate programs (23.1%, *n* = 509), other tuition and stipend programs (20.7%, *n* = 456), high school programs (20.6%, *n* = 454), NRMN programs (11.6%, *n* = 255), and postdoctoral programs (11.3%, *n* = 250).

**Table 3.** Responses to prior mentorship or research program measure

Prior mentorship or research program (*n* = 2,204)		
**Program**	***n***	**%**
Research training	1,355	61.5
Other summer research program	1,018	46.2
Other career advancement programs	861	39.1
Academic Advising and Support	778	35.3
Program during post-graduate or graduate	578	26.2
Program during undergraduate	509	23.1
Other tuition and stipend program	456	20.7
Program during high school	454	20.6
NRMN programs	255	11.6
Program during post-doc	250	11.3

Respondents were asked to select all programs that apply. Percentages are based on the total number of respondents who selected at least one program (*n* = 2,204). Full survey items are provided in Supplementary table 1.

Subgroup analyses by career stage, gender, and combined race/ethnicity were conducted as illustrative examples of the types of exploratory analyses that can be performed using this dataset. These analyses provide insights into patterns of prior mentorship and research program participation across diverse groups. Detailed results tables for these subgroup analyses are provided in the supplementary materials for reference. It is important to note that the data for these subgroups include only respondents who provided both demographic information and prior mentorship or research program participation, which may influence the representativeness of the findings within each subgroup.

Analysis by career stage revealed variation in the prior mentorship or research program experience reported, as would be expected from participants engaging and having engaged with different experiences at different career stages. For each group, we highlight key participation trends. Seventy-nine percent (*n* = 998) of the 1,264 faculty members who indicated prior program participation reported participating in research training programs, including workshops, field experiences, and conferences. Graduate students (*n* = 76) participated in research training programs (76.3%, *n* = 58) and summer research programs (64.5%, *n* = 49), along with other types of programs. Postdoctoral fellows (*n* = 175) were involved in research training programs (61.1%, *n* = 107) and academic advising and support programs (46.3%, *n* = 81). Medical students (*n* = 186) showed participation in summer research programs (63.4%, *n* = 118). While undergraduate students (*n* = 485) who indicated prior program participation selected fewer program types, they engaged in high school research programs (27.2%, *n* = 132) and academic advising and support programs (37.1%, *n* = 180). A few cases did not fully align with the participants’ reported career stage. For example, three undergraduate students indicated participation in graduate or postgraduate programs. These responses were retained in the dataset, and the original frequencies are provided in the supplementary materials. Such instances highlight potential inconsistencies that researchers should be aware of when analyzing the data.

In terms of gender, both male (*n* = 699) and female respondents (*n* = 1,415) reported similar patterns of involvement in prior mentorship or research programs. The most common involvement for both genders was in research training programs (69.1%, *n* = 483 for males; 57.7%, *n* = 816 for females) and summer research programs (51.6%, *n* = 361 for males; 43%, *n* = 609 for females). Career advancement programs were also similarly reported by both groups, with 39.9% (*n* = 564) of female respondents and 37.9% (*n* = 265) of male respondents participating. Respondents identifying outside of the binary gender (*n* = 24) engaged in research training programs (58.3%, *n* = 14) and academic advising and support programs (54.2%, *n* = 13).

In terms of race/ethnicity, the most frequently reported programs among White respondents (*n* = 1,050) were research training programs (76%, *n* = 798) and summer research programs (55.1%, *n* = 579). They also participated in career advancement programs (47.3%, *n* = 497). Hispanic respondents (*n* = 272) took part in research training programs (61.8%, *n* = 168) and summer research programs (44.1%, *n* = 120), with some involvement in NRMN programs (16.9%, *n* = 46). Black respondents (*n* = 590) were most frequently involved in academic advising and support programs (34.6%, *n* = 204) and high school programs (23.3%, *n* = 138). They reported lower participation in other mentorship and research programs. Asian respondents (*n* = 328) engaged in research training programs (68.6%, *n* = 225) and summer research programs (53.7%, *n* = 176). Respondents from other racial/ethnic groups (*n* = 120) were involved in research training programs (65.8%, *n* = 79) and summer research programs (54.2%, *n* = 65).

## Discussion

While other national datasets have also focused on various training and education efforts in the sciences, efforts usually collect data at the program (rather than the individual) level [27,28], or have focused on specific career stages such as undergraduates or faculty [29–34]. The multi-study common measures dataset collected across the 11 studies in NRMN Phase II, therefore, present a unique opportunity to examine the long-term efficacy of mentorship interventions and their impact on the career development and persistence of researchers across career stages (undergraduate through faculty). By leveraging common measures across the studies, we were able to harmonize the data, enabling us to assess NRMN’s impact beyond each individual study. With over 6,000 participants spanning diverse backgrounds, career stages, and programs, this large dataset holds the potential for future study into the collective impact of the NRMN initiative.

Due to the multi-study nature of the common measures dataset, the included data come from studies that vary in sample sizes, recruitment methods, and specific measures administered. This variation, along with differences in missing data and the subsets of demographic and program participation variables collected by each study, results in uneven group sizes and incomplete overlap across measures. Consequently, direct comparison of participation patterns across demographic groups is challenging, and aggregation into unified figures risks oversimplifying or misrepresenting the data. For this reason, we present detailed tables to provide transparency and preserve the integrity of the data. Nonetheless, the narrative highlights key trends and disparities to support interpretation of the findings.

### Demographic representation

Seventy percent (70%) of participants in NRMN Phase II identified as women, and 46% identified as White. Many studies have noted the disproportionate participation of women in career and professional development programs in higher education [35–39]. Participants across racial/ethnic groups, including Black and Asian participants, were similarly represented in this sample compared to national data on academic science workforce demographics [40,41]. Additionally, 91.4% of participants did not report a disability. This prevalence aligns with national statistics on the disability prevalence among academic scientists [42–43]. While the representation of many scientists in this paper reflects broader trends in the field, there remains an opportunity to develop more inclusive and accessible career and professional development pathways to better support scientists from varied socio-cultural identities throughout their careers [4].

### Mentor training participation

Around 60% of the participants in NRMN Phase II reported prior experience with mentor training, indicating familiarity with mentorship education before engaging in an NRMN intervention. While most studies did not collect data on the specific mentor training interventions participants had previously received, the five-year span of NRMN Phase II overlapped with heightened focus on mentor training nationally, including requirements from both the National Science Foundation (NSF) [44] and across the National Institutes of Health (NIH) [45], which may have influenced the high proportion of mentors with prior training. The increasing emphasis on mentorship education in these agencies may reflect a growing recognition of the importance of mentorship in the success of early-career scientists.

### Exposure to career and professional development interventions

Data on exposure to various career and professional development prior to the NRMN interventions revealed some variation across demographic groups. While participation in such programs was relatively widespread, women and Black scientists appeared to have slightly lower rates of engagement when adjusted for their total representation within the dataset. Notably, more than half of the Black participants in the dataset were from a single study that specifically targeted first-year undergraduate students at Historically Black Colleges and Universities (HBCUs). This focus on freshmen likely contributed to the higher participation of Black participants in high school programs, where they showed the highest involvement compared to other racial/ethnic groups. However, outside of high school programs, Black participants had lower prior participation in other mentorship and research programs. This pattern may not be fully representative of the broader Black scientific community. It is important to note that the sample sizes (n’s) are not comparable across groups, which may affect the interpretation of these results. Future work is required to test whether these differences in exposure to mentorship and research programs are statistically significant, whether they reflect broader trends or are specific to the study population, and if they are unique to the population of participants who engaged in the NRMN interventions.

### Potential for future research

Beyond demographic and background data, the NRMN Phase II dataset includes rich information on psychosocial and career outcomes, collected from over 15,000 baseline and follow-up surveys. This dataset offers substantial potential to answer critical questions regarding the collective impact of NRMN interventions on the career trajectories of scientists from diverse backgrounds. While the current analysis is descriptive, future research leveraging these data will provide valuable insights into how mentorship influences long-term career success and persistence across diverse groups in STEMM (Science, Technology, Engineering, Mathematics, and Medicine).

The data from NRMN Phase II can contribute to advancing the science of mentorship and further the goals of exploring the impact of mentorship practices on career outcomes for diverse groups in biomedical research. The commitment made by the NIH through the DPC and the NRMN initiative provided a foundation upon which future mentorship programs can be built, ensuring that scientists from all backgrounds have access to the support they need to thrive.

## Limitations of study

This study has several limitations, primarily stemming from the design and scope of NRMN Phase II. The NRMN CC played a central role in compiling common measures data and facilitating collective research activities but had limited involvement in overseeing study-specific processes, such as survey design, data management, or analysis. As a result, variability in data collection and reporting practices across studies introduced inconsistencies and presented unique challenges at the multi-study level. Additionally, due to IRB protocols, the NRMN CC was unable to mandate the sharing of participant contact information, limiting the ability to track data at the individual level.

The NRMN Phase II studies included different intervention strategies and study designs, including active interventions, control groups, and waitlist groups, across both cross-sectional and longitudinal frameworks [25,17]. These distinctions are important because categorizing the data based on these groups could reveal differences in outcomes and participant experiences. However, this paper focuses on describing participant characteristics and prior training experience based primarily on baseline survey data, prior to any intervention. Furthermore, recruitment strategies varied across studies and were often tailored to specific populations, shaping the composition of the overall NRMN participant sample.

Other limitations include the reliance on self-reported data, which is susceptible to social desirability and recall bias [16,24] Data incompleteness and differences in survey items and scales used across the studies also present challenges for data synthesis and interpretation. Finally, some inconsistencies in demographic classifications, such as participants selecting multiple categories for mutually exclusive items, may affect the consistency of the findings.

Despite these limitations, the large dataset collected across the 11 NRMN studies offers incredible potential to gain critical insights for future research on the long-term impact of mentorship on STEMM careers. While the inherent challenges must be considered, the data present important opportunities to explore and deepen our understanding of mentorship’s role in the professional development of biomedical scientists from diverse backgrounds.

## Future directions and recommendations

The inclusion of individuals from diverse backgrounds is crucial for advancing the biomedical research workforce and fostering innovation within the scientific community. The NRMN was established to support career development across groups, with a strong emphasis on mentorship and professional development. This paper provides a descriptive analysis of the rich data collected across the NRMN Phase II studies. The dataset includes data from an array of common measures, which could offer critical insights into the factors influencing career progression and mentorship effectiveness and inform the pathways to success for diverse groups in biomedical research.

One key direction for future research is to conduct large-scale analyses of aggregated data from multiple cohorts and interventions across career stages. This approach enables the identification of patterns and correlations that may not be evident when analyzing individual studies. These analyses could provide a deeper understanding of how specific mentorship models and career development practices influence outcomes for individuals from different backgrounds, including how demographic variables (e.g., career stage, race, gender, field of study, and institutional affiliation) shape mentorship experiences and success. Statistical analyses using demographic and contextual factors could uncover areas where mentorship strategies are less or more effective for specific groups, highlighting opportunities for targeted interventions. This would directly support the NRMN’s overarching goal of advancing inclusive, accessible, and equitable mentorship practices in biomedical research, helping to ensure that mentorship is a transformative and empowering experience for all individuals, regardless of background.

In addition to demographic considerations, incorporating longitudinal data into future analyses is crucial for understanding the long-term effectiveness of mentorship interventions. This could include examining existing longitudinal data collected by the studies or by tracking career outcomes and effectiveness over extended periods to assess whether certain models produce lasting benefits and how the trajectories of underrepresented scientists evolve beyond the immediate post-intervention phase.

Finally, comparative analyses with other large datasets in the field would provide critical context for interpreting the findings from this study. By evaluating the strengths and limitations of the NRMN common data in comparison to others, researchers can identify potential biases and gaps, which could inform the development of improved methodologies and data collection practices. This comparative approach would not only enhance the validity of current findings but also guide future research by highlighting areas where additional data or methodological refinements are needed.

In conclusion, the NRMN multi-study common measures dataset provides one of the most comprehensive and diverse collections of mentorship data in biomedical research, offering a unique opportunity to examine how mentorship influences the success of diverse groups. By addressing the limitations identified in this study and expanding on the future directions outlined above, subsequent research can deepen our understanding of mentorship’s role in supporting biomedical scientists. Future studies that embrace more inclusive, longitudinal, and comparative approaches will ultimately contribute to refining mentorship practices and fostering a more equitable biomedical research workforce.

## Supporting information

10.1017/cts.2025.10133.sm001 Hyun et al. supplementary material Hyun et al. supplementary material

## Supplementary material

The supplementary material for this article can be found at https://doi.org/10.1017/cts.2025.10133.

## Previous presentations

Poster presentation at Translational Science 2025, held in Washington, D.C. from April 14–17, 2025.
