# Characteristics of participants in the national research mentoring network studies – ERRATUM

**DOI:** 10.1017/cts.2025.10167

**Published:** 2025-10-14

**Authors:** So Hee Hyun, Emma Dums, Fátima Ruiz Sancheznieto, Kimberly Spencer, Julie M. Hau, Jenna Griebel Rogers, Christine Pfund

**Affiliations:** Institute for Clinical and Translational Research (ICTR), University of Wisconsin–Madison, Madison, WI, USA

When this article was originally published in the Journal of Clinical and Translational Science it included an extra piece of supplementary material. This was included in error and has since been removed.

The publisher apologises for this error.
