# Characteristics of participants in the national research mentoring network studies – CORRIGENDUM

**DOI:** 10.1017/cts.2026.10714

**Published:** 2026-03-06

**Authors:** So Hee Hyun, Emma Dums, Fátima Sancheznieto, Kimberly Spencer, Julie M. Hau, Jenna Griebel Rogers, Christine Pfund

When this article was originally published in the Journal of Clinical and Translational Science it contained an error in the name of the author Fátima Sancheznieto. This has been updated in the original article.

The authors apologise for this error.
