# Supplementary material for: Characteristics of participants in the national research mentoring network studies
Source: J Clin Transl Sci. 2025 Aug 26;9(1):e204. doi: 10.1017/cts.2025.10133 (PMC12505240; doi:10.1017/cts.2025.10133)
Supplement: Hyun et al. supplementary material [file S2059866125101337sup001.docx]

**Supplementary table 1.** Full survey items of prior mentorship or research program

| **Have you ever been a part of any of the below programs focusing on research training? (Select all that apply)** |
| --- |
| - Program with research training component during HIGH SCHOOL |
| - Program with research training component during UNDERGRADUATE years such as MARC, RISE, NSF-REU, BUILD |
| - Program with research training component during POST-GRADUATE or GRADUATE years such as IMSD, PREP |
| - Program with research training component during POST-DOC years such as IRACDA |
| - NRMN programs (MyMentor, MyNRMN, webinars, etc.) |
| - Other tuition and stipend program |
| - Other summer research program (i.e,. Summer Research Experience) |
| - Academic Advising and Support (i.e., tutoring, STEM advising, additional math courses, etc.) |
| - Research training (i.e., workshop, field experience, conferences, etc.) |
| - Other career advancement programs (i.e., networking, GRE Prep, field trips, career panels, etc.) |

**Supplementary Table 2.** Responses to prior mentorship or research program measure by career stage (*n* = 2,198)

| **Prior mentorship or research program: Have you ever been a part of any of the below programs focusing on research training? (Select all that apply)** | | | | | | | | | | | | |
| --- | --- | --- | --- | --- | --- | --- | --- | --- | --- | --- | --- | --- |
|  | **Faculty**  **(*n* = 1,264)** | | **Undergrad. student**  **(*n* = 485)** | | **Graduate student**  **(*n* = 76)** | | **Medical student**  **(*n* = 186)** | | **Postdoctoral**  **Fellow**  **(*n* = 175)** | | **Other**  **(*n* = 12)** | |
| **Program** | ***n*** | **%** | ***n*** | **%** | ***n*** | **%** | ***n*** | **%** | ***n*** | **%** | ***n*** | **%** |
| Program during high school | 257 | 20.3 | 132 | 27.2 | 7 | 9.2 | 37 | 19.9 | 17 | 9.7 | 2 | 16.7 |
| Program during undergraduate | 279 | 22.1 | 66 | 13.6 | 33 | 43.4 | 74 | 39.8 | 53 | 30.3 | 2 | 16.7 |
| Program during post-graduate or graduate | 494 | 39.1 | 3 | 0.6 | 14 | 18.4 | 21 | 11.3 | 44 | 25.1 | 1 | 8.3 |
| Program during post-doc | 228 | 18.0 | 0 | 0.0 | 0 | 0.0 | 1 | 0.5 | 21 | 12.0 | 0 | 0.0 |
| NRMN programs | 190 | 15.0 | 31 | 6.4 | 2 | 2.6 | 0 | 0.0 | 31 | 17.7 | 1 | 8.3 |
| Other tuition and stipend program | 306 | 24.2 | 59 | 12.2 | 13 | 17.1 | 12 | 6.5 | 65 | 37.1 | 0 | 0.0 |
| Other summer research program | 687 | 54.4 | 102 | 21.0 | 49 | 64.5 | 118 | 63.4 | 55 | 31.4 | 5 | 41.7 |
| Academic Advising and Support | 432 | 34.2 | 180 | 37.1 | 36 | 47.4 | 47 | 25.3 | 81 | 46.3 | 0 | 0.0 |
| Research training | 998 | 79.0 | 90 | 18.6 | 58 | 76.3 | 87 | 46.8 | 107 | 61.1 | 11 | 91.7 |
| Other career advancement programs | 613 | 48.5 | 145 | 29.9 | 41 | 53.9 | 8 | 4.3 | 47 | 26.9 | 4 | 33.3 |

The table includes only data from respondents who had both career stage and prior mentorship or research program information (*n* = 2,198), excluding participants who did not have data for both measures.

Respondents were asked to select all that apply for prior mentorship or research program.

Percentages are based on the number of respondents of each career stage.

Full survey items are provided in Supplementary table 1.

**Supplementary Table 3.** Responses to prior mentorship or research program by gender (*n* = 2,133).

| **Prior mentorship or research program: Have you ever been a part of any of the below programs focusing on research training? (Select all that apply)** | | | | | | |
| --- | --- | --- | --- | --- | --- | --- |
| **Program** | **Female (*n* = 1,415)** | | **Male (*n* = 699)** | | **Other (*n* = 24)** | |
|  | ***n*** | **%** | ***n*** | **%** | ***n*** | **%** |
| Program during high school | 303 | 21.4 | 132 | 18.9 | 7 | 29.2 |
| Program during undergraduate | 308 | 21.8 | 164 | 23.5 | 10 | 41.7 |
| Program during post-graduate or graduate | 317 | 22.4 | 245 | 35.1 | 4 | 16.7 |
| Program during post-doc | 140 | 9.9 | 104 | 14.9 | 0 | 0.0 |
| NRMN programs | 176 | 12.4 | 75 | 10.7 | 0 | 0.0 |
| Other tuition and stipend program | 295 | 20.8 | 145 | 20.7 | 5 | 20.8 |
| Other summer research program | 609 | 43.0 | 361 | 51.6 | 9 | 37.5 |
| Academic Advising and Support | 515 | 36.4 | 226 | 32.3 | 13 | 54.2 |
| Research training | 816 | 57.7 | 483 | 69.1 | 14 | 58.3 |
| Other career advancement programs | 564 | 39.9 | 265 | 37.9 | 6 | 25.0 |

The table includes only data from respondents who provided both gender and prior mentorship or research program information (*n* = 2,133), excluding participants who did not respond to both questions.

Percentages are based on the number of respondents of each reported gender.

“Other” gender responses include the common measure responses of non-binary, transgender, and other.

The common measures in this table are both multi-select measures.

Full survey items are provided in Supplementary table 1.

**Supplementary Table 4.** Responses to prior mentorship or research program by combined race/ethnicity (*n* = 2,140).

| **Prior mentorship or research program: Have you ever been a part of any of the below programs focusing on research training? (Select all that apply)** | | | | | | | | | | |
| --- | --- | --- | --- | --- | --- | --- | --- | --- | --- | --- |
|  | **Asian**  **(*n* = 328)** | | **Black or African American (*n* = 590)** | | **Hispanic, Latinx, or Spanish origin**  **(*n* = 272)** | | **White**  **(*n* = 1,050)** | | **Other**  **(*n* = 120)** | |
| **Program** | ***n*** | **%** | ***n*** | **%** | ***n*** | **%** | ***n*** | **%** | ***n*** | **%** |
| Program during high school | 72 | 20.0 | 138 | 23.4 | 35 | 12.9 | 213 | 20.3 | 26 | 21.7 |
| Program during undergraduate | 92 | 28.0 | 94 | 15.9 | 83 | 30.5 | 237 | 22.6 | 25 | 20.8 |
| Program during post-graduate or graduate | 88 | 26.8 | 54 | 9.2 | 77 | 28.3 | 369 | 35.1 | 40 | 33.3 |
| Program during post-doc | 37 | 11.3 | 34 | 5.8 | 27 | 9.9 | 152 | 14.5 | 21 | 17.5 |
| NRMN programs | 24 | 7.3 | 68 | 11.5 | 46 | 16.9 | 132 | 12.6 | 9 | 7.5 |
| Other tuition and stipend program | 61 | 18.6 | 107 | 18.1 | 70 | 25.7 | 230 | 21.9 | 27 | 22.5 |
| Other summer research program | 176 | 53.7 | 146 | 24.7 | 120 | 44.1 | 579 | 55.1 | 65 | 54.2 |
| Academic Advising and Support | 92 | 28.0 | 204 | 34.6 | 104 | 38.2 | 396 | 37.7 | 45 | 37.5 |
| Research training | 225 | 68.6 | 169 | 28.6 | 168 | 61.8 | 798 | 76.0 | 79 | 65.8 |
| Other career advancement programs | 103 | 31.4 | 173 | 29.3 | 90 | 33.1 | 497 | 47.3 | 48 | 40.0 |

The table includes only data from respondents who provided both combined race/ethnicity and prior mentorship or research program information (*n* = 2,140), excluding participants who did not respond to both questions.

Percentages are based on the number of respondents of each reported race/ethnicity.

Full survey items are provided in Supplementary table 1.

The common measures presented in this table are both multi-select measures.
